# Supplementary material for: A dual-mode mobile phone microscope using the onboard camera flash and ambient light
Source: Sci Rep. 2018 Feb 19;8:3298. doi: 10.1038/s41598-018-21543-2 (PMC5818495; doi:10.1038/s41598-018-21543-2)
Supplement: Supplementary file 8 — Supplementary Information [file 41598_2018_21543_MOESM8_ESM.pdf]

Supplementary Information: A dual-mode mobile phone microscope using the onboard camera flash and ambient light

A. Orth,[a] E. R. Wilson [a], J. G. Thompson[b] and B. C. Gibson[a]

[a]ARC Centre of Excellence for Nanoscale BioPhotonics, School of Science, RMIT University, Melbourne, Australia

[b]ARC Centre of Excellence for Nanoscale BioPhotonics, Robinson Research Institute, School of Medicine, The University of Adelaide, Adelaide, Australia

Caco-2 sample preparation

A culture of human colon colorectal cells Caco-2 , HTB-37™ (ATCC , Manassa, VA, USA) was maintained using the ATCC standard culture method for this cell line. Briefly, the cells were incubated at 37˚C with 5% CO_2 in Dulbecco's Modified Eagle Media (DMEM, Gibco™, USA) containing 100 U/mL penicillin-streptomycin and 10% fetal bovine serum (FBS, Sigma-Aldrich, USA). Subculture was performed when cells were 80% confluent by using 0.25% Trypsin (Sigma-Aldrich, USA) for 5-10 minutes at 37˚C and centrifugation at 200 x g at 4˚C for 5 minutes.

Low passage number Caco-2 cells were grown on coverslips at a density of 2 x 10^5 cells per cover glass, using the same conditions as above and incubated overnight (18 hours). Cells were incubated at room temperature (19 ˚C±1) for 10 minutes with 10% formalin neutral buffered solution (Sigma-Aldrich, USA). The cells were washed three times with phosphate buffered saline pH7 (PBS, Gibco™, USA) before mounting in a PBS solution containing 10% Glycerol (Sigma-Aldrich, USA).
